# Factors influencing adherence to antiretroviral therapy from the experience of people living with HIV and their healthcare providers in Sierra Leone: a qualitative study

**DOI:** 10.1186/s12913-022-08606-x

**Published:** 2022-11-08

**Authors:** Michael Lahai, Sally Theobald, Haja R. Wurie, Sulaiman Lakoh, Patrick O. Erah, Mohamed Samai, Joanna Raven

**Affiliations:** 1 Faculty of Pharmaceutical Sciences, College of Medicine and Allied Health Sciences, University of Sierra Leone, Freetown, 00232 Sierra Leone; 2 Department of International Public Health, Liverpool School of Tropical Medicine, Liverpool, UK; 3 College of Medicine and Allied Health Sciences, University of Sierra Leone, Freetown, Sierra Leone; 4 Faculty of Pharmacy, University of Benin, Benin City, Nigeria

**Keywords:** Family, Community and health system support, Antiretroviral therapy adherence

## Abstract

**Background:**

Antiretroviral therapy (ART) is the primary mode of treatment for Human Immunodeficiency Virus (HIV). It slows disease progression and reduces the spread of infection. HIV treatment is also known to require a high level of adherence of over 90% to achieve good treatment outcomes and viral load suppression. In Sierra Leone, about 70% of People Living with HIV (PLHIV) are non-adherent in their first year of treatment. Understanding the reasons behind this high rate of non-adherence from the perspectives of both PLHIV and health workers is critical for developing strategies to improve adherence. This qualitative study is rooted in the field of public health services. It identifies the barriers and facilitators influencing adherence to antiretroviral treatment in Sierra Leone.

**Methods:**

A qualitative study design using in-depth interviews of four healthcare workers and 16 PLHIV in two districts in Sierra Leone– Freetown and Bo. The interviews were analyzed using a grounded theory approach to identify emerging themes from the data.

**Results:**

The study identified several facilitators and barriers to ART adherence at the personal, community, and health system levels. The facilitators included perceived benefits of ART, family support, having an informal caregiver, receiving free ART medicines, and belonging to peer support groups. The identified barriers were stigma and discrimination, frequency of medication, use of traditional medicine, lack of money for food and transport, work barriers, inadequate medicines and test kits, limited health workers, and long distances to clinics.

**Conclusions:**

Our study emphasized the need for implementing behavioural change communication programmes and activities to reduce stigma and discrimination in the community. Knowledge of the facilitators and barriers to antiretroviral therapy could provide relevant information for more responsive and equitable programmes supporting adherence implementation in low- and middle-income countries. This study also identifies the vital need for community integration of HIV treatment services.

**Supplementary Information:**

The online version contains supplementary material available at 10.1186/s12913-022-08606-x.

## Background

The global commitment to Fast-Track the Human Immunodeficiency Virus (HIV) response and end Acquired Immunodeficiency Disease Syndrome (AIDS) by 2030 is not on track [1]. The recent Covid-19 lockdowns in 2020 led to a reduction of HIV testing by 41% and referrals for diagnosis and treatment by 37% compared to similar data in 2019 [2]. In 2019, almost 700,000 AIDS-related death and 1.7 million new HIV infections were reported [2]. Ending AIDS as a global public health threat in low- and middle-income countries may require a yearly increase of about 35% in HIV spending without inflation [2, 3]. The 90–90-90 target by the Joint United Nations Program for HIV/AIDS aims at interventions to achieve 90% viral suppression. Achieving this high level of viral load suppression requires that 90% of people living with HIV/AIDS (PLHIV) know their status, and 90% diagnosed with HIV are receiving ART [4, 5]. Systematic reviews and adherence studies revealed that about 20% of people living with HIV do not consistently use the medication in low- and middle-income countries(LMICs) [6–9]. In Sierra Leone, 78,000 people were living with HIV in 2019, and 48% of PLHIV are aware of their status, of which only 43% are receiving ART [1].

The treatment for HIV/AIDS is a combination of antiretroviral drugs. ART stops HIV from multiplying and can suppress HIV to undetectable levels in the blood. Thus allowing a person’s immune system to recover, overcome infections, prevent the development of AIDS, and reduce the risk of HIV transmission [10–12]. Adherence describes how a person uses and receives treatment according to medical recommendations, including timing, dosing, and consistency. A qualitative study in Sierra Leone revealed that addressing the barriers of socioeconomic and psychological factors may help prevent loss to health facility follow-up [13]. PLHIV with better socioeconomic status related to income, education, and employment status were more likely to adhere to treatment than those with poor socioeconomic status [14]. Barriers include medication and health concerns, stigma, family responsibilities, and problems with schedule and routine [15]. The expansion and decentralization of HIV/AIDS services could improve health services at the community level, increases collaboration through task sharing between health professionals, improve access to treatment and overcome the burden of limited human resources in hospitals [16]. Other studies revealed that ART initiation intervention among PLHIV who are more likely to drop out of treatment could improve adherence [17]. A cohort study in Sierra Leone showed that 62% of eligible ART patients stopped care before initiating ART [18]. The main barriers in this cohort study include a lack of understanding due to poor relationships and communication between people living with HIV and healthcare professionals.

This study also recognized the need for context-specific interventions to support adherence that considers access to medicines, care, and support for people living outside the capital city of Freetown. A Better understanding of the barriers and facilitators around adherence to ART in Sierra Leone from both PLHIV and their healthcare providers is critical to developing interventions that support ART adherence. This study will provide relevant information for decision-makers, public health professionals, and clinicians on implementing more responsive and equitable ART programs to improve adherence.

## Methods

### Study site

**Fig. 1:**
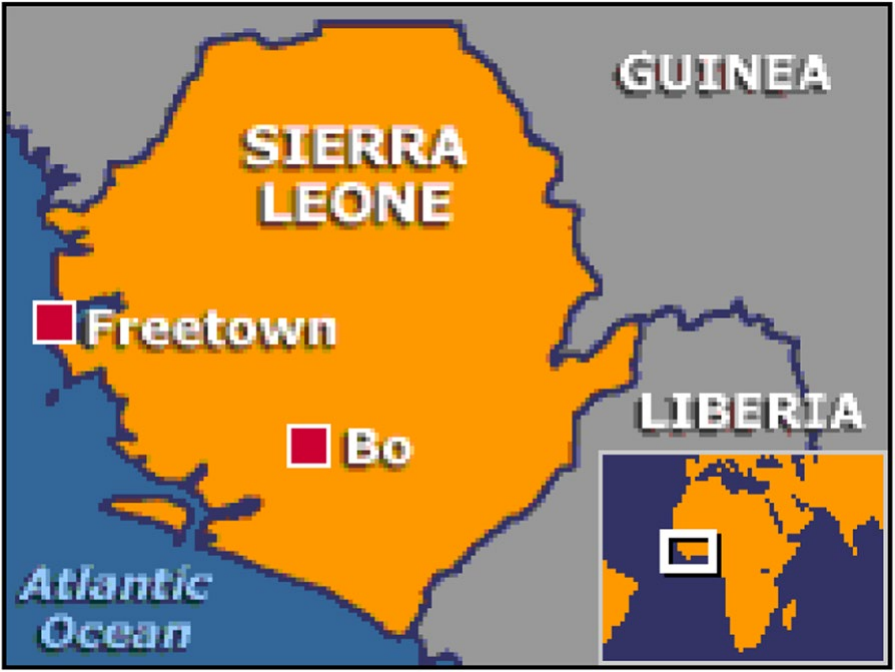
Map of Sierra Leone showing the selected study sites

The study was conducted in the two main referral hospitals in Sierra Leone: Connaught Hospital in the capital city of Freetown and the Bo government hospital in Bo, the second major city in Sierra Leone (see Fig. 1). Freetown and Bo districts were deliberately chosen as they are the two districts with the highest prevalence of HIV/AIDS in Sierra Leone, with rates of 2.7 and 1.8 in Freetown and Bo, respectively [19]. Connaught hospital is a 300-bed hospital that houses an HIV/AIDS clinic and ward. It also serves as the main referral center for adult HIV/AIDS care in Freetown. Bo government hospital is a 500-bed hospital with a clinic and ward for PLHIV.

### Study design and sampling

A qualitative study design employing in-depth interviews was used to explore the barriers and facilitators to adherence to and provision of ART [20]. Qualitative interviews generate in-depth and contextual information about an individual’s experiences, beliefs, and perceptions and explore the reasons behind their answers through probing questions [21]. In-depth interviews with four healthcare workers and sixteen people living with HIV were employed to explore the complexities of the issues related to ART adherence. The sample size for health workers is small and reflects the small number of healthcare professionals providing care for PLHIV in the selected clinics. The research adopted a triangulation strategy, including PLHIV and healthcare workers’ views on ART adherence, to enhance the integrity of the findings [22].

- Purposive sampling was used to select participants based on features or characteristics that will enable a detailed understanding of the topic [23]. The criteria for inclusion were: PLHIV who were taking ART for the last six months
- PLHIV who registered for ART but stopped treatment in the past six months
- Patient aged 18 years and above and spoke English or Krio.
- Healthcare workers involved in the care of PLHIV.

Exclusion criteria were:

- PLHIV, who do not attend the HIV clinic at the Bo Government Hospital and Connaught Hospital.
- PLHIV aged less than 18 years.
- Health workers not involved in the care of PLHIV

### Data collection

Interviews with people living with HIV were done by the lead author, in the Krio language, in a private room in the HIV clinic at Connaught Hospital and the Bo Government Hospital, and lasted for 54 min. These interviews commenced on the 26^th^ of February 2018 to the 16^th^ of March 2018. The topic guide for PLHIV was used to explore the following (see topic guides in Additional file 1. Appendix):

1. Participants’ background,
2. their experience with ART,
3. the barriers and enablers to using ART services,
4. other related services
5. concerns about taking ART.

It took time to build rapport with the respondents so that they could talk openly about their experiences. The lead author sensitively asked the questions and observed signs of discomfort or distress. The lead author interviewed the health workers in English for 60 minutes in their office. The topic guide for Healthcare workers was used to explore the following (Additional file 1. Appendix 1):

1. participants’ background
2. their roles and responsibilities in ART provision,
3. their perception and experiences of adherence to ART, and
4. their role in improving adherence to ART.

At the end of the interview, participants were compensated with three united states dollars ($) to reimburse for any transport costs and their time.

### Data analysis

The recordings were transcribed verbatim. Where necessary, the recordings were transcribed in the local language (Krio) and then translated into English. A sub-set was checked against the recordings for the quality of transcription and translation. The interviews were analyzed using the grounded theory approach [24]. The framework analysis was determined by an inductive conceptualization [25], with data managed using Nvivo 11 programme. A coding framework was developed based on the areas explored in the interviews and on themes emerging from the data (see Additional file 1. Appendix 2 for the coding framework). The coding framework was applied to transcripts of all interviews. Charts were then developed for each theme, and these charts were used to describe the themes.

## Results

### Participant characteristics

Sixteen PLHIV were interviewed, with eight from Bo and eight from Freetown. The majority were female [11], adherent to ART (nine), and employed (nine). There were more female participants in Bo, and in Freetown, most participants had employment (Table 1). Four health workers were interviewed, with two from each study site. Three health workers were female (Table 1).

**Table 1:** characteristics of study participants (PLHIV and healthcare workers)

**Study site**	**Adherence to ART**		**Gender**		**Age (years)**			**Work**	
	**Adherent**	**Non-adherent**	**Female**	**Male**	**24–35**	** > 35–45**	** > 45**	**employed**	**Unemployed**
Bo	5	3	7	1	3	3	2	3	5
Freetown	4	4	4	4	4	3	1	6	2
	**Type of health worker**		**Gender**		**Age (years)**		-	-	-
	Clinician	Counsellor	Female	Male	20–30	30–40	-	-	-
Bo	1	1	2	-	1	1	-	-	-
Freetown	1	1	1	1	-	2	-	-	-

### Facilitators and barriers to adherence to ART

**Fig. 2:**
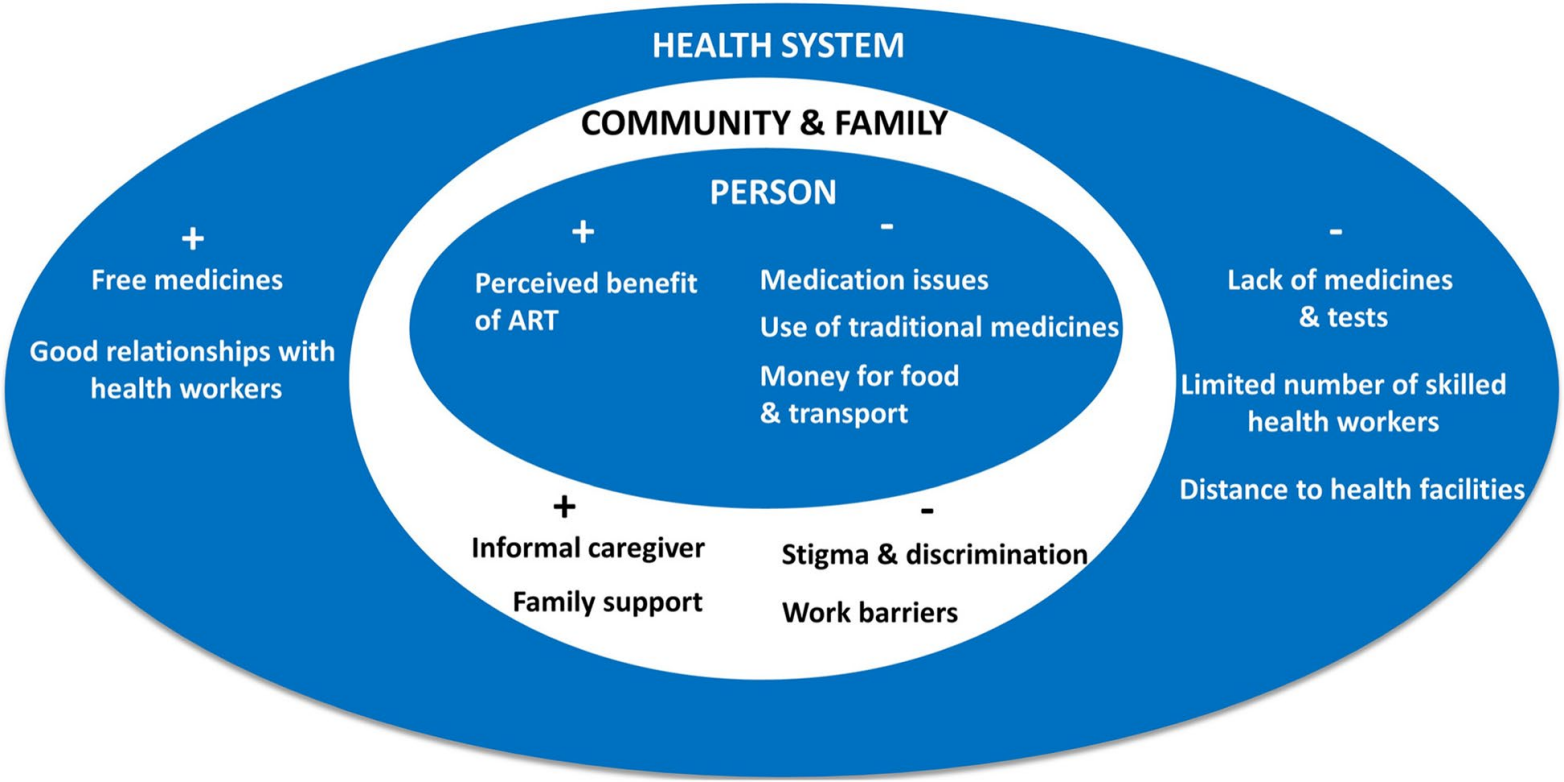
Facilitators and barriers to ART adherence (+ indicates facilitators;—indicates barriers)

The study revealed several facilitators and barriers to ART adherence at personal, family and community, and health system levels (Fig. 2). These facilitators and barriers are now described along with illustrative quotations from the study respondents.

### Personal level

#### Perceived benefits of ART

Most participants were optimistic about the effects of ART, especially when they could take the ART medication as prescribed and had regular appointments for follow-up. They explained that HIV care had improved their quality of life and health status.

“I felt perfect when I started taking medicine when I was confirmed HIV positive. Currently, I am not feeling good and losing weight […] I was informed at the treatment centre that my blood is low, and I believe it happened because I missed my medicine pick-up appointment. I have started treatment and am beginning to feel great again.”. (Woman, non-adherent, Bo).

#### Medication issues

Some PLHIV reported that the taste and size of the medicines deterred them from adhering to the treatment regime. Healthcare workers expressed concerns about the prescribed frequency of taking ART medicines.

“It’s challenging to take a drug every day of your life, especially with the kind of pills and the daily intake that is required coupled with other additional blood medicines” (Woman, Health worker, Bo).

“For me, it is the big tablet […] when I started taking the medicine at first, because of the taste, I [would] usually vomit” (Woman, Adherent, Bo)

#### Use of traditional medicines

Some PLHIV reported feeling better and stopped taking their ART, believing that traditional herbal medications would provide a long-lasting solution. However, they eventually became seriously ill.

“It was last year that I stopped taking the medication. I was asked to retake the HIV test this year when I started feeling sick. I informed the healthcare professionals that I was using herbal medicine to treat HIV” (Man, non-adherent, Freetown).

#### Money for food and transport

Many PLHIV reported that they found it difficult to maintain gainful employment due to their illness which affected their acquisition of food needed when taking ART. It also made it difficult for them to pay for transportation to the health facilities. They relied on friends or relatives to provide food and help with transport but often felt embarrassed to ask for help.

“The little money I had is no more, and there is nothing left at the moment […] if they will help with supply or money […] when I regain my health, I will be able to start my business and support my children” (Woman, Non-Adherent, Freetown).

All healthcare workers reported that many PLHIV did not continue their treatment because they lacked food or money for transportation. They also reported that food provided was for PLHIV who were underweight.

“They will say I do not take the medications because there is no food […] others will say i do not have transport […] we have a system for providing food for people with low body mass index, and that is all at the moment” (Woman, health worker, Freetown).

### Family and community level

#### Informal caregiver

Many PLHIV and healthcare workers ascertained the importance of having an informal caregiver at home that can help with adherence to ART.

“It is good for someone living with HIV to have an informal caregiver” (Woman, Health worker, Freetown).

The informal caregiver can remind the PLHIV to take the medication at different times of the day, encourage them to eat healthy food, and attend the clinic*.*

“I informed my younger sister that the medication was for prevention, and she must remember me to take my medicine in the morning and at night. She is informing me daily.” (Woman, adherent, Bo).

#### Family support

Some PLHIV and all health workers reported that support from family members is essential. They can provide practical support such as picking up medication, reminders about taking it, attending clinic appointments, and supporting travel to the clinic. They also provide psychological support to help them to be optimistic about their future.

“We need to pass the message to the family and relatives so that they will be able to support these people. If you stigmatize PLHIV, they will lose hope for survival. Family is important because they spend about 90–95 percent of their time with them (Male Health worker, Freetown). Imagine the man is negative, and the wife is positive. Nevertheless, he is here to collect the medication for his wife.” (Woman, health worker, Bo).

#### Stigma and discrimination

PLHIV reported stigma and discrimination from family, friends, and community members because of their positive HIV status. They explained that HIV/AIDS is a taboo subject and, as such, their condition should not be discussed or disclosed to others.

“Yes, initially I was staying with my husband’s relatives […]when they discovered that I had the virus […] they started laughing at me […] they started throwing provocative words at me, they don’t even give me food to eat and they finally asked me out of the house (crying) […] Just look at me, my hair is not even neat because I don’t have someone to plait my hair” (Woman, adherent, Bo).

Some PLHIV explained that the community members associate frequent ailments and weight loss with HIV and reject them. However, when they start ART and gain weight, they are accepted.

“Initially, my friends were not properly talking to me because I had lost weight from the disease […]. Now they feel free to talk and interact with me. Thanks to God for that […]. I did not inform them about my condition […] they would have been afraid of me if I had informed them of my status” (Woman, adherent, Bo).

Healthcare workers also reported many examples of PLHIV experiencing stigma and discrimination, such as lack of support, fear of death, and not sharing toilets and items such as cups and cutlery. They also confirmed that PLHIV could not access and adhere to ART, thereby profoundly affecting their mental health.

“Look at this lady; she came this morning crying […]. She is staying with her mother, constantly reminding her that she will die soon from HIV/AIDs. She is crying […] She said, if my mum is not ready to support me, how could I survive?” (Woman, health worker, Freetown).

Many PLHIV reported that they were frightened of disclosing their HIV status to their partners for fear of their relationship ending. One woman explained that her husband gave her money and sent her back to her village after disclosing her status.

“I was taken to the hospital, where my husband received the test and informed me that I was HIV positive. I did not believe him initially. He said we could not continue the relationship. He said that I may have contracted the disease from my work as a hairdresser (used needle and comb). He, therefore, asked me to return to Bo (my hometown) and gave me Le150,000 (£15 or $19) as hospital costs. At the hospital in Bo, I was tested and confirmed again to be HIV positive. I was encouraged to commence medication and advised to meet the requirement of a monthly appointment” (Woman, adherent, Bo).

Many PLHIV reported not disclosing their status to other members of their family. Others said they would avoid discussing their HIV status with anyone because they could be harassed or ostracized.

“It has to be kept secret. We were advised not to disclose it because people will start pointing fingers secretly (He/She is HIV positive). They also advise us to take our medications” (Woman, adherent, Bo).

Health workers also reported that many PLHIV prefers not to disclose their status to their families and are prepared to keep their medication and condition a secret.

“Most of our patients do not give us the correct information about their partner […] they usually say; my husband is not here, my husband has travelled[…]when it is time for that person(maybe married) to take their medication in the presence of their partner, it is a problem” (Woman, health worker, Freetown).

#### Work barriers

PLHIV reported improvement in their health when they adhered to ART. However, many found it challenging to take the medicines regularly when working and often missed their medication during work hours.

“the medication is good because if you take medication for a specific illness and see improvement, it is good [….] I miss my medication when I go out to work” (Woman, adherent, Bo).

PLHIV faced discrimination in the workplace; a woman spoke about losing her job because her employer observed her taking medicine at the same time every day.

“they are watching me […] I was informed that If I know that I have a disease and I am taking a drug, let me be careful […]when it’s time to take the drug, I would think of my life, and my only daughter […]I would usually take the drug. Later, I was called and dismissed from my duty.” (Woman, non-adherent, Freetown).

### Health system

#### Free medicines

The health workers emphasized the importance of ensuring that ART continues to be accessible for PLHIV in LMIC, as the high cost of these drugs would prohibit uptake and adherence by PLHIV.

“Regarding the fight against HIV, the medication should be free. If the medication is free, it will help most of our clients[…]the cost of HIV medication is too high” (Woman, health worker, Freetown).

#### Good relationships with health workers

PLHIV reported a good relationship with healthcare workers involved in their treatment. They explained that health workers encourage them to take their medications and attend the clinic. They talk to them about their illness and situation, providing comfort and reassurance.

“They treat me well, and they also encourage me. I got discouraged on my first day at the hospital, but the nurse talked to me and encouraged me” (Woman, adherent, Bo).

#### Limited numbers of skilled health workers

Health workers were concerned about the limited number of health staff working against PLHIV. They felt much pressure to provide care and support and could not spend enough time with PLHIV. They believed there was a need for more staff that could increase the time spent on assessing, reviewing, and supporting PLHIV.

“I will love to have another staff that would help us to assess patient (Woman,health worker, Bo).

#### Lack of medicines and tests

Healthcare workers reported a lack of ART medicines and test kits used in caring for PLHIV. They explained that donour organizations provide drugs and test kits and frequent hospital procurement delays.

“…in terms of HIV care you go to any health facility there is the issue of drug stock out and [….]test kits shortage” (Woman, health worker, Bo).

#### Distance to health facilities

PLHIV reported that they had long distances to travel to the ART clinics. In Bo, people travel at least 30 min, costing the equivalent of $1.5. In Freetown, people travel for one hour to the clinic, costing about $2.5 from the farthest distance.

“They need to bring the health facility close to my community if possible because there is no health facility where I live” (Woman, adherent,, Freetown).

## Discussion

The study revealed several facilitators and barriers to ART adherence at three levels-personal, family and community, and health system. The main findings of the study are:

*On a Personal level*, most PLHIV revealed that adherence to ART and treatment follow-up improved their quality of life. Adherence augmentation programs by healthcare professionals should be implemented as an essential program for patients and/partners by trained professionals. Thus, ensuring that their patients understand the benefit of ART adherence by understanding the common side effects, the long-term positive effects of daily and continual use, and its association with improved quality of life and prevention of HIV transmission. In this study, PLHIV also expressed medication-related adherence challenges regarding the size of the tablet and the required daily intake of ART. Several studies showed that Pharmacist interventions through appointments was vital in the preventing and reducing of drug-related problems and improving patient adherence to ART with subsequent boost to their immune system [26–29]. Our study revealed concerns about the lack of money for food or transportation to the hospital. Other studies also confirmed that transportation cost to healthcare facilities could impede daily adherence (missed doses) to ART and lead to significant patient follow-up loss [30]. This study revealed the need for interventions that improve access and adherence to ART medication.

*On the Family and community support* level, the four [4] healthcare workers in this study and PLHIV revealed that support from close family members and having an informal caregiver was important in improving adherence among PLHIV. PLHIV also confirmed that it was essential to have an informal caregiver to support and ensure adherence. Other studies revealed that non-disclosure of positive HIV status to a partner could lead to HIV transmission, treatment discontinuity, poor health outcomes and difficulty in HIV prevention and control [31–33]. Healthcare workers and patients mentioned the availability of feeding programs in communities among peer support groups to encourage interaction and adherence among people receiving ART. Several studies reaffirmed the importance of implementing adherence programs that provide enough information to aid long-term adherence for PLHIV in peer groups other than as individuals [34–37].

In addition, this study revealed several issues of stigmatization among extended family members. Other research findings suggested an existing gap in perception between PLHIV, healthcare professionals and community individuals. They emphasized the need for improvement of such interactions [38] through the stepping stone approach that improves the knowledge of the community by using mass media campaigns and direct contact or testimonies from PLHIV [39–41].

*Health systems support level*: Our study confirmed the need to recruit additional skilled and motivated staff to support treatment and ensure that medicines and test kits are free and always available for PLHIV. Thus, patient confidence and reliance in the health sector reduce loss to follow-up at health facilities. A study in Uganda revealed that there was a significant association between missing doses and missing appointments. Therefore, it is important to ensure that strategies are designed to help improve ART medication adherence among PLHIV. The key to any strategy is to ensure that, in addition to adherence measures, ART medications should be free and available at all times because about 60% of the population in Sierra Leone are poor [42] and may not be able to pay for treatment or ART medications. A study in Sierra Leone also suggested that interventions that address socioeconomic and psychological factors may help reduce disengagement from ART initiation [14]. Adherence to ART could be improved by integrating treatment services by using accredited pharmacies [43] and clinics [44] close to communities. Such improvement may help reduce spending on transportation for PLHIV, reduce long-distance hospital visits for medication refills while improving adherence monitoring, interaction and collaboration between the primary healthcare facilities and hospitals.

### Limitations

It was a sensitive topic to explore, especially in an environment where stigma and discrimination towards PLHIV are common, and PLHIV may have been reluctant to express their ART experiences. By establishing a good rapport and sensitive questioning, we hope to have minimized this. In addition, PLHIV may have concealed information related to adherence because the interviews were conducted at the HIV clinic, where they also receive treatment. This was minimized by ensuring that no one entered the room during the interviews and by removing all personal identifiers from the transcripts and reports.

## Conclusions

Our study revealed that several factors influence adherence. The factors identified as facilitators are the perceived benefit of ART by PLHIV, family and community support, informal caregiver, free medicine, and good relations with health workers. The barriers include stigma and discrimination, medication frequency, and health-seeking behaviours.

These health-seeking behaviours include:

- traditional medicines use
- lack of money for food and transport
- lack of medicines and test kits, limited healthcare workers (medical doctors, pharmacists, and nurses), and
- distance to the health facility.

Strengthening personal, community, and family support and health system support level will help PLHIV to continue ART, improve access to HIV care, reduce loss to treatment follow-up and prevent or reduce stigmatization in the community.

## Supplementary Information

**Additional file 1.**

## Data Availability

All data and materials are available, and can be provided upon reasonable request to the corresponding author.

## Abbreviations

PLHIV: People living with Human Immunodeficiency virus
HIV/AIDs: Human Immunodeficiency Virus/Acquired Immunodeficiency Virus
ART: Antiretroviral therapy
CD4: Cluster of differentiation 4
LMIC: Low and middle-income country
