# Supplementary material for: Factors influencing adherence to antiretroviral therapy from the experience of people living with HIV and their healthcare providers in Sierra Leone: a qualitative study
Source: BMC Health Serv Res. 2022 Nov 8;22:1327. doi: 10.1186/s12913-022-08606-x (PMC9644013; doi:10.1186/s12913-022-08606-x)
Supplement: Supplementary file 1 — Additional file 1. [file 12913_2022_8606_MOESM1_ESM.docx]

*Appendix 1*

Table A: characteristics of participants (healthcare professionals-HCP)

| **No.** | **ID** | **District** | **Hospital** | **Gender** | **Age (Yrs)** |
| --- | --- | --- | --- | --- | --- |
| 1 | HCPB1 | Bo | Bo Government(B) | Female | 20- 30 |
| 2 | HCPB2 | Bo | Bo Government(B) | Female | 30- 40 |
| 3 | HCPC2 | Western Area | Connaught | Male | 30- 40 |
| 4 | HCPC1 | Western Area | Connaught | Female | 30- 40 |

 C: Connaught hospital; B: Bo Government Hospital

Table B: characteristics of participants (People living with HIV-PLHIV)

| **No.** | **ID** | **District** | **Adhering to ART (yes or No)** | **Gender** | **Age (Yrs)** |
| --- | --- | --- | --- | --- | --- |
| 1 | PB6AF | Bo | Adherent(A) | Female(F) | 24- 35 |
| 2 | PB7NF | Bo | Non-adherent | Female | 24- 35 |
| 3 | PB2AF | Bo | Adherent | Female | >35- 45 |
| 4 | PB1AF | Bo | Adherent | Female | >35- 45 |
| 5 | PB5NF | Bo | Non-adherent | Female | 24- 35 |
| 6 | PB3AF | Bo | Adherent | Female | >45 |
| 7 | PB4AF | Bo | Adherent | Female | >45 |
| 8 | PB8NF | Bo | Non-adherent(N) | Male(M) | >35- 45 |
| 9 | PC8NF | Freetown | Non-Adherent | Female | >35- 45 |
| 10 | PC7AF | Freetown | Adherent | Female | 24- 35 |
| 11 | PC1NF | Freetown | Non-adherent | Female | 24- 35 |
| 12 | PC2NM | Freetown | Non-adherent | Male | 24- 35 |
| 13 | PC3AM | Freetown | Adherent | Male | >35- 45 |
| 14 | PC4AF | Freetown | Adherent | Female | >35- 45 |
| 15 | PC5NM | Freetown | Non-adherent | Male | 24- 35 |
| 16 | PC6AM | Freetown | Adherent | Male | >45 |

 C: Connaught hospital; B: Bo Government Hospital; P: patient

*Appendix 1*

**Topic Guide 1: Exploring the factors influencing adherence to antiretroviral therapy amongst people living with HIV/AIDS in Sierra Leone: a qualitative study.**

**Topic guide: In depth interview with PLHIV who are not adhering to ART**

**Objectives:**

- To explore the demographic factors of PLHIV.
- To identify the factors that facilitate and hinder adherence to ART.
- To understand the reasons for complying or not complying with appointment for hospital visit amongst PLHIV.
- To describe their relationships with family, friends and the community members and how this affect adherence to ART

**Introduction:** Introduce the project, the scope of the interview

**Informed Consent Process:** Ensure participant has read the information sheet, ask if she / he has any questions or areas for clarification, explain about confidentiality including recording the interview, complete consent sheet.

**Details of participant:**

| Interviewee ID |  | Job |  |
| --- | --- | --- | --- |
| Date of Interview |  | Gender | Male □ Female □ |
| Time of start of interview |  | District |  |
| Time of end of interview |  | Facility name |  |
| Name of interviewer |  | Age |  |
| Name of transcriber |  |  |  |

1. **Background**

- I would like to know a little bit more about you. What do you do as a job? What family do you have? Where do you live?
- When were you first diagnosed with HIV?
- Have you ever been advised/encouraged by a health worker/social worker/people providing care and support services/association of PLHIV to start on ART?

1. **Awareness of ART**

- What do you know about ART? (Probe on: What is ART? What are the benefits, side-effects, rumors, realities, etc?)
- Do you know where people can obtain ART in this area? (Probe on: paid services, free access, facilities—-government, private, accessibility, direct and indirect costs.)
- Do you know or do you have friends/families who are currently taking ART? (Probe on: how did they start ART? Did they talk to you about it? Did they advise you to take ART?)

1. **Experience of ART**

- What is your experience of ART? (Probe on: improved health outcome, adverse effects, pill burden, lack of food, lifestyle issues, adherence, etc.)
- How do you describe your health situation before and after ART?
- Does your family know about you taking ART? Do your close friends know you are on ART? Why and why not?
- What support is available for you in the community, in the family, in the workplace? (Probe on: care and support services, discrimination, and stigma.)

1. **Reasons for discontinuing ART**

- Why did you discontinue ART? (Probe on: stigma, do not think it is beneficial, discrimination, fear of side effect, do not know where to go, lack of family supportive, poverty, transport problems, etc.)
- What did you do after discontinuation? (Probe on: treatment options.)
- How do you compare your health situation before and after discontinuation?
- Do you want to restart on treatment? What do you want to see happening for you to restart ART?
- What do you think could be done to help people like you to continuously use ART and properly adhere to treatment?

1. **Possible barriers**

- How does your community view people who are taking ART? (Probe on: stigma, discrimination, supportive attitude, negative attitude, etc.)
- Do people taking ART face any socioeconomic and other problems because of taking ART? (Probe on: poverty, hunger, transport problems, lack of support, etc.)
- What do you think would be the reaction of your family/friends if you start taking ART? Why and why not? (Probe on: separately for family and friends perceived reaction.)

1. **Existing programme activities related to ART**

- What activities take place at the moment in your community to help PLHIV Existing program activities on access ART? (Probe on: awareness programs, health facilities, NGOs, home-based care ART givers, associations of PLHIV, etc.)
- What should be done to ensure that PLHIV access ART in this community? (Probe on: the role of government, NGOs, facilities, associations

1. **Is there anything else you would like to add?**

**Closure and thank you.**

**Topic guide 2: Exploring the factors influencing adherence to antiretroviral therapy amongst people living with HIV/AIDS in Sierra Leone: a qualitative study.**

**Topic guide: In depth interview with PLHIV who are adhering to ART**

**Objectives:**

- To explore the demographic factors of PLHIV.
- To identify the factors that facilitate and hinder adherence to ART.
- To understand the reasons for complying or not complying with appointment for hospital visit amongst PLHIV.
- To describe their relationships with family, friends and the community members and how this affect adherence to ART

**Introduction:** Introduce the project, the scope of the interview

**Informed Consent Process:** Ensure participant has read the information sheet, ask if she / he has any questions or areas for clarification, explain about confidentiality including recording the interview, complete consent sheet.

**Details of participant:**

| Interviewee ID |  | Job |  |
| --- | --- | --- | --- |
| Date of Interview |  | Gender | Male □ Female □ |
| Time of start of interview |  | District |  |
| Time of end of interview |  | Facility name |  |
| Name of interviewer |  | Age |  |
| Name of transcriber |  |  |  |

1. **Background**

- I would like to know a little bit more about you. What do you do as a job? What family do you have? Where do you live?
- When were you first diagnosed with HIV?
- When did you first start on ART? Or when were you first advised to start ART?

1. **Experience of ART**

- What do you understand by the term ART?
- What is your experience of ART? (Probe on: improved health outcome, adverse effects, pill burden, lack of food, lifestyle issues, adherence, etc.)
- How do you describe your health situation before and after ART?
- Does your family know about you taking ART? Do your close friends know you are on ART? Why and why not?
- What support is available for you in the community, in the family, in the workplace? (Probe on: care and support services, discrimination, and stigma.)

1. **Process of getting ART**

- Tell me about how you get your ART (get them to describe the process: how frequently they visit clinic, what happens at the clinic, who they see, how long do they stay at the clinic, how far they have to travel etc)
- How do you think you are being treated (handled) by the health workers? (Probe on: in relation to adherence: privacy, confidentiality, respect, being listened to, time spent with patient, waiting time, integration with other services, etc.)
- What do you think about the counselling that you receive? (Probe on: especially importance of adherence)
- What do you think could be done to help people like you to adhere more easily to their treatment?
- Do you know PLHIV on ART that discontinued treatment? (Probe on: why did they discontinue? Economic problem, side effect, stigma, etc.)

1. **Concerns about taking ART**

- What do you perceive as the biggest problem regarding taking ART?
- What do you think could be done to improve this?

1. **Existing programme activities related to ART**

- What activities take place at the moment in your community to help PLHIV Existing program activities on access ART? (Probe on: awareness programs, health facilities, NGOs, home-based care ART givers, associations of PLHIV, etc.)
- What should be done to ensure that PLHIV access ART in this community? (Probe on: the role of government, NGOs, facilities, associations

1. **Is there anything else you would like to add?**

**Closure and thank you.**

**Questionnaire 3: Exploring the factors influencing adherence to antiretroviral therapy amongst people living with HIV/AIDS in Sierra Leone: a qualitative study.**

**Topic guide: In depth interview with health care worker**

**Objectives:**

- To explore the perception and workload of Healthcare workers involved in the treatment of PLHIV.
- To explore the social, economic, cultural, health and health system factors affecting adherence to ART in two districts in Sierra Leone.
- To identify recommendations for the development of strategies to improve adherence to ART.

**Introduction:** Introduce the project, the scope of the interview

**Informed Consent Process:** Ensure participant has read the information sheet, ask if she / he has any questions or areas for clarification, explain about confidentiality including recording the interview, complete consent sheet.

**Details of participant:**

| Interviewee ID |  | Job title and cadre of health worker |  |
| --- | --- | --- | --- |
| Date of Interview |  | Gender | Male □ Female □ |
| Time of start of interview |  | District |  |
| Time of end of interview |  | Facility community name |  |
| Name of interviewer |  | Age |  |
| Name of transcriber |  |  |  |

1. **Background of health worker**

- What is your job in the health sector? How long have you been in this job? How long have you worked here?

1. **Role and responsibilities in ART provision**

- I would like to know more about your work in providing ART. What is your role in providing ART to PLHIV? How long have you been doing this?
- In a normal work day, what do you do?
- What do you enjoy about this work? What do you least enjoy?

1. **Challenges in ART provision**

- What are the challenges that you face in providing ART services
- Probe about: number of patients, number of staff, availability of drugs, availability of tests

1. **People’s adherence to ART**

- I would like to know about adherence to ART. How do you think your patients do, generally speaking, in terms of adherence to ART? (Probe on: Do your patients keep their appointments? Why and why not?)
- Which groups of patients in this facility are more or less likely to adhere to ART? (Probe on: age, sex, economic status, educational status, marital status, religion, etc.)
- What are the main challenges you face in supporting your patients to adhere to ART?
- What are the most common reasons for people not adhering to ART use in this community?
- Probe on:
  - Lack of knowledge
  - Poverty / Economic constraints: costs of transport; loss of income when travelling; seeking employment
  - Stigma and discrimination of being HIV positive
  - Social support
  - Family pressures and responsibilities
  - Religion
  - Having a good diet – being able to pay for this
  - Side effects of drugs
- Among the people who do adhere to ART, what do you think helps people adhere?
  - Social support
  - Family support
  - Peer groups
  - Health worker counselling and support

1. **Improve adherence to ART**

- What have you done to help improve adherence to ART? Are there any guidelines in the hospital on promoting adherence? If so, can I see them
- What else could be done to help people better adhere to ART?
- How do you think those could be implemented in this hospital?
- If you could choose one recommendation that is the most important, what would that be and why?

1. **Is there anything else you would like to add?**

**Closure and thank you.**

Appendix 2: Code book for PLHIV and Healthcare workers

1. Background
   1. Reliability of the test result
   2. Reactions to diagnosis
   3. Disclosure of HIV status
   4. Daily work activity
2. Experience of ART
   1. Death from ART non-Compliance
   2. Understanding of ART
   3. Experience with taking ART
   4. Stigmatization of HIV or ART
   5. Socioeconomic Problems with ART
   6. Description with Health Situation before and after ART
3. Concerns about taking ART
   1. Mental Health of PLHIV
   2. Possible reasons for HIV transmission
   3. Recommendations for improving patient adherence
   4. Medication related problems
   5. Possible reasons for discontinuation of treatment
4. Existing program activities related to ART
   1. Recommendations for government, NGO’s, facilities and associations in improving adherence
   2. Support to health Centers and patients
   3. Knowledge of community programs for improving access to ART
5. Provision of ART
   1. Role in the provision of ART
   2. Recommendations for improving access to ART
   3. Good relations
   4. Privacy and confidentiality
   5. Challenges in provision of ART
   6. Waiting time, length of discussion in the clinic and frequency of visit
   7. Understanding of counselling sessions
